# Establishment of an immortalized stromal cell line derived from human Endometriotic lesion

**DOI:** 10.1186/s12958-020-00669-x

**Published:** 2020-11-23

**Authors:** Zhi-Xiong Huang, Rong-Feng Wu, Xiao-Mei Mao, Shao-Min Huang, Tian-Tian Liu, Qiong-Hua Chen, Qing-Xi Chen

**Affiliations:** 1 School of Life Sciences, Xiamen University, Xiamen, 361102 China; 2 Reproductive Medical Center, The First Affiliated Hospital of Xiamen University, Xiamen, China; 3 The Key Laboratory of Research and Diagnosis of Gynecological Diseases of Xiamen City and Department of Obstetrics and Gynecology, the First Affiliated Hospital of Xiamen University, Xiamen, 361003 China

**Keywords:** Immortalized, Endometriotic stromal cells, Estrogen, Inflammation, Tumorigenicity

## Abstract

**Background:**

Endometriosis is a benign gynecological disease with obviously feature of estrogen-dependence and inflammatory response. The applications of primary endometriotic stromal cells in research of endometriosis are restricted for short life span, dedifferentiation of hormone and cytokine responsiveness. The objective of this study was to establish and characterize immortalized human endometriotic stromal cells (ihESCs).

**Methods:**

The endometriotic samples were from a patient with ovarian endometriosis and the primary endometriotic stromal cells were isolated from the endometriotic tissues. The primary cells were infected by lentivirus to establish telomerase reverse transcriptase (hTERT)-induced immortalized cells. Quantification of mRNA and proteins was examined by quantitative real-time polymerase chain reaction (qRT-PCR) and Western Blot. CCK-8 assay and EdU labeling assay were assigned to assess the growth of ihESCs. Karyotype assay was performed to detect the chromosomes of ihESCs. Colony formation assay and nude mouse tumorigenicity assay were used to evaluate colony-formation and tumorigenesis abilities.

**Results:**

ihESCs continuously overexpressed hTERT via infection of lentivirus and significant extended the life span reaching 31 passages. The morphology, proliferation and karyotype of ihESCs remained unchanged. The expression of epithelial-mesenchymal transition (EMT) markers, estrogen-metabolizing proteins and estrogen/progesterone receptors (ERs and PRs) were unaltered. Furthermore, the treatment of estrogen increased the proliferation and EMT of ihESCs. Lipopolysaccharides (LPS) and IL-1β remarkably induced inflammatory response. The clonogenesis ability of ihESCs was consistent with primary cells, which were much lower than Ishikawa cells. In addition, nude mouse tumorigenicity assay demonstrated that ihESCs were unable to trigger tumor formation.

**Conclusion:**

This study established and characterized an immortalized endometriotic stromal cell line that exhibited longer life span and kept the cellular morphology and physiological function as the primary cells. The immortalized cells remained normal feedback to estrogen and inflammatory response. Moreover, the immortalized cells were not available with tumorigenic ability. Therefore, ihESCs would be serviceable as in vitro cell tool to investigate the pathogenesis of endometriosis.

## Introduction

Endometriosis, a frequent gynecology disease of reproductive-aged female, is defined that the endometrium is present outside of the uterus location [1, 2]. The estimated 10% of women within childbearing age may suffer from endometriosis [3, 4]. And appearance of endometriosis generally accompanied by chronic pelvic pain, dysmenorrhea, severe dyspareunia, and even infertility [5–7]. It is approximated that 47% of infertile women have endometriosis [8]. Moreover, the high recurrence rate of endometriosis required three or more surgeries [9, 10]. Therefore, endometriosis negative impacts on the large number of women patients and indirectly on healthcare systems and society.

Although many factors reported were closely associated with incidence of endometriosis, the etiology of this disease is still unknown. Of all pathogenic theories about endometriosis occurrence, hypothesis of retrograde menstruation is the most recognized version, in which retrograde menstruation of endometrial tissue slough through patent fallopian tubes into the peritoneal cavity and then adhere, invade and proliferate in ectopic locations [2]. These processes are concerned with background of family heredity, hormone response, and immunological factors [11–13].

Endometriosis is reputed as estrogen and inflammation-dependent disease [14, 15]. It was reported that the increased level of aromatase in eutopic endometrial tissues allowed the implantation of ectopic tissues on peritoneal surfaces [16]. Capellino et all claimed that estrogen, via its functional receptors, modulated macrophages to regulate the immune response [17]. And Han et al. found that ERβ inhibited TNF-alpha-induced apoptosis and promoted inflammation by increasing IL-1β to enhance cellular adhesion and proliferation of ectopic endometriotic tissues [18]. Thus, the stimulation of estrogen and inflammation are mutually beneficial during establishment of endometriotic lesions.

For investigations on the molecular mechanisms of endometriosis, cultured human primary endometriotic cells are desirable in vitro tools. However, the applicability of primary endometriotic cells is largely restricted by dedifferentiation during cell culture, including loss of hormone and cytokine responsiveness, and short life span. The subjective of this study was to establish and characterize an hTERT-induced immortalized endometriotic stomal cell line (ihESCs), which kept primary morphology and proliferation. The expression of EMT markers, estrogen synthetase and estrogen/progesterone receptors in ihESCs remained unchanged. Moreover, ihESCs contained normal number of chromosomes and were confirmed not maligned transformed by tumorgenicity assay.

## Materials and methods

### Reagents

DMEM/F12 medium (#SH30023.01) was from Hyclone (Shanghai, China) and fetal bovine serum (FBS; #04–001-1A) was bought from Biological Industries (BI; Cromwell, CT, USA). 17β-estradiol/estrogen/E2 (#E2758) and lipopolysaccharides (LPS; #L4391) were from Sigma-Aldrich (Shanghai, China). Recombinant Human IL-1β (#C600002) was bought from Sangon Biotech (Shanghai, China). GAPDH mouse monoclonal antibody (#40493) was procured from ABclonal (Boston, USA). Cytokeratin 18 antibodies (#4548), E-cadherin antibodies (#14472), Vimentin antibodies (#5741), N-cadherin antibodies (#13116), Progesterone Receptor (PR) antibodies (#8757S), and ERα antibodies (#13258) were acquired from Cell Signaling Technology (Danvers, MA, USA). HSD17B1 antibodies (#ab51045), TERT antibodies (#ab32020), ERβ antibodies (#ab288), COX-2 antibodies (#ab15191) and CYP17A1 antibodies (#ab125022) were purchased from Abcam (Cambridge, UK).

### Patients and primary cells culture

Endometriotic tissues were collected in Department of Obstetrics and Gynecology in the First Affiliated Hospital of Xiamen University. The application of samples received permission from the ethics committee and all patients signed the informed consent. Endometriotic lesions were from a 35-year-old female suffered from ovarian endometriosis confirmed by laparoscopy and histopathology. This endometriosis patient had regular menstrual cycles and was without hormone treatment for more 3 months before the surgery.

The endometriotic samples were minced and digested by collagenase IV (#A004186–0001; Sangon Biotech; Shanghai, China) and deoxyribonuclease I (DNase I; #B002004–0005; Sangon Biotech; Shanghai, China). After being filtered through nylon cell-strainers with a 100 mesh which intercepted pieces of tissue, and then through a mesh size of 400 which blocked the epithelial glands cells and passed through the stromal cells. The epithelial and stromal cells were respectively cultured within DMEM/F12 medium containing 10% FBS in dishes at 10% CO_2_ 37 °C incubation.

### Establishment of ihESCs

hTERT, human telomerase reverse transcriptase, activates telomerase activity which maintains telomere length and extends cell lifespan [19]. hTERT coding regions were cloned and inserted into CV186 vector (GeneChem; Shanghai, China) and then mixed with lentivirus packing plasmids (VSVG/PMDL/REV). The mixture of plasmids was transfected into 293 T cells by Lipofectamine 3000 (#L3000015; ThermoFisher; Shanghai, China) to generate lentivirus. The primary endometriotic stromal cells were cocultured with viral supernatants for 24 h to generate infected cells.

### Drugs treatment

5 × 10^5^ cells were plated into 60 cm dishes in DMEM/F12 medium without phenol red. After 24 h culture to remove endogenous hormones, 200 nM estrogen, 100 μg/ml LPS or 10 μg/ml IL-1β were respectively diluted in DMEM/F12 medium with 10% charcoal-treated FBS (#04–201-1A; Cromwell, CT, USA) and exposed to cells for 48 h.

### RNA extraction and qRT-PCR

The lysis of cells was performed by RNAiso Plus (#9108; Takala Biotechnology; Kyoto, Japan) and total RNA was extracted by chloroform and isopropyl alcohol. The synthesis of cDNA was by using PrimeScript RT reagent kit (#RR047A; Takara). PCR reaction was executed by the SYBR Premix Ex Taq II (#RR820A, Takala) in Lightcycler 480 (Roche; Basel, Switzerland). The results were normalized based on GAPDH expression and calculated by the 2^-△△CT^ method. All primer sequences were listed in Table 1.

**Table 1:** Primer sequences used in qRT-PCR analysis

Gene Name	Sequences (5′-3′)
GAPDH	Forward: GGAAGGTGAAGGTCGGAGTCA Reverse: GAGTCCTTCCACGATACCAA
hTERT	Forward: CGGCCTATTCCCCTGGT Reverse: ATGTTCCTCCCAGCCTTGA
IL-1β	Forward: ATGATGGCTTATTACAGTGGCAA Reverse: GTCGGAGATTCGTAGCTGGA
IL-6	Forward: ACTCACCTCTTCAGAACGAATTG Reverse: CCATCTTTGGAAGGTTCAGGTTG
IL-10	Forward: TCAAGGCGCATGTGAACTCC Reverse: GATGTCAAACTCACTCATGGCT
MCP-1	Forward: AGTGTCCCAAAGAAGCTGTGA Reverse: CCTGAACCCACTTCTGCTTG

### Western blot assay

Total proteins were harvested by RIPA lysis buffer with protease inhibitors. SDS-PAGE was assumed to separate proteins of different molecule mass which then were transferred onto PVDF membranes (#IPVH00010; Merck; Germany). The PVDF membranes were incubated within primary antibodies for overnight at 4 °C. After cleaned by PBS, the PVDF membranes were then immersed in corresponding secondary antibody. The dilution rates of antibodies were hTERT (1:1000), Cytokeratin 18 (1:1000), E-cadherin (1:500), Vimentin (1:1000), N-cadherin (1:1000), COX-2 (1:1000), HSD17B1 (1:500), CYP17A1 (1:1000), ERα (1:1000), ERβ (1:500), PR (1:1000), GAPDH (1:10000), and secondary antibody (1:10000). The signal was finally visualized by an enhanced chemiluminescence (ECL; # NCI4106; Pierce, Waltham, MA, USA).

### Observation of cell ultrastructure

A total of 1 × 10^5^ cells were planted into 35 cm dishes which of bottom were polylysine-coated cover slides. After 24 h till all cells attached to the slides, 2.5% glutaraldehyde and 1% osmic acid were employed to fix the cells. After dehydrated with gradient ethanol, the slides were immersed in tert-butanol overnight at 4 °C. And the slides were freeze-dried and then sprayed with gold. The images of cell ultrastructure were observed and photographed by Scanning Electron Microscope (#JSM-6390LV; Japan Electron Optics Laboratory; Tokyo, Japan).

### Cell proliferation assay

To analyze the proliferation of cells, Cell Counting Kit-8 (#HY-K0301; MedChemExpress; Shanghai, China) was used to quantify the growth rate. 5 × 10^3^ cells were planked into 96-well plates and treated with 200 nM estrogen. CCK-8 was added into each well and reacted in 37 °C incubator for 2 h every 24 h. The absorbance of supernatants was measured by an ELISA reader spectrophotometer (Dynatech Laboratories, Chantilly, VA).

### EdU labeling assay

According to the instructions of EdU assay kit (#C0071S; Beyotime Biotechnology, Shanghai, China), 1 × 10^5^ ihESCs were seeded into 6-well plates and treated with 200 nM estrogen for 48 h. The treated cells were incubated with 10 μM EdU solution for 3 h. After fixed and permeabilized, the cells were cultured in click sdditive solution for 30 min. Finally, Hoechst 33342 solution was employed to stain the cell nucleus. The results were observed and captured using the fluorescence microscope (Olympus Corporation, Tokyo, Japan).

### Karyotype analysis

1 × 10^6^ cells were planted into in a 10 cm dish with DMEM/F12 medium containing 10% FBS for 48 h. After incubated with 0.2 μg/ml colchicine (#HY-16569; MCE; Shanghai, China) for 2 h, the cells were treated with low osmotic pressure for 20 min and then fixed by fixative solution (methanol: acetic acid =3:1). The fixed cells were added to the slide and stained by Giemsa. The images of chromosomes observed and captured by microscope were clipped and sorted by Photoshop soft.

### Immunocytochemistry (ICC)

Primary cells and ihESCs were planked on 6-well plates and cultured to 100% confluence. The cells were fixed by cold methanol and permeated by 0.3% Triton-X. After incubated with 5% bovine serum albumin (BSA) for 1 h, the cells were covered within primary antibody overnight. The immunocomplexes were incubated with corresponding secondary antibody for 1 h at room temperature. DAB Color Development Kit (#AR1022; Boster Biological Technology, Wuhan, China) was used to stain the immunocomplexes. The results of immunostaining were captured using the microscope (Olympus Corporation, Tokyo, Japan).

### Colony formation assay

A total of 1 × 10^3^ primary cells, ihESCs or Ishikawa cells were evenly planked in 6 cm dishes with DMEM/F12 containing 10% FBS. After cultured for 7 days, the colonies of cells were wash with PBS and fixed by 4% paraformaldehyde for 10 min. 0.1% crystal violet (#E607309; Sangon Biotech; Shanghai, China) was applied to stain the cells for 15 min. The colonies were photographed and counted visually.

### Xenograft

The acquisition of female BALB/c nude mice (5–8 weeks old and 15–20 g) and operation were relied on Animal Research Laboratory of Xiamen University. The ethics of animal experiment was strictly followed in the course of experiments. The mice were acclimatized for a week before processing. The mice were divided into 3 groups and each group contained 3 mice. The primary ESCs, ihESCs and Ishikawa cells were subcutaneously injected into the right dorsal of the mice (1 × 10^7^ cells/mouse) according to grouping. The mice were monitored for enough tumor growth. After 2 months, the mice were sacrificed and photographed.

## Results

### Establishment of hTERT-induced immortalized endometriotic stromal cells by infection of lentivirus

**Fig. 1:**
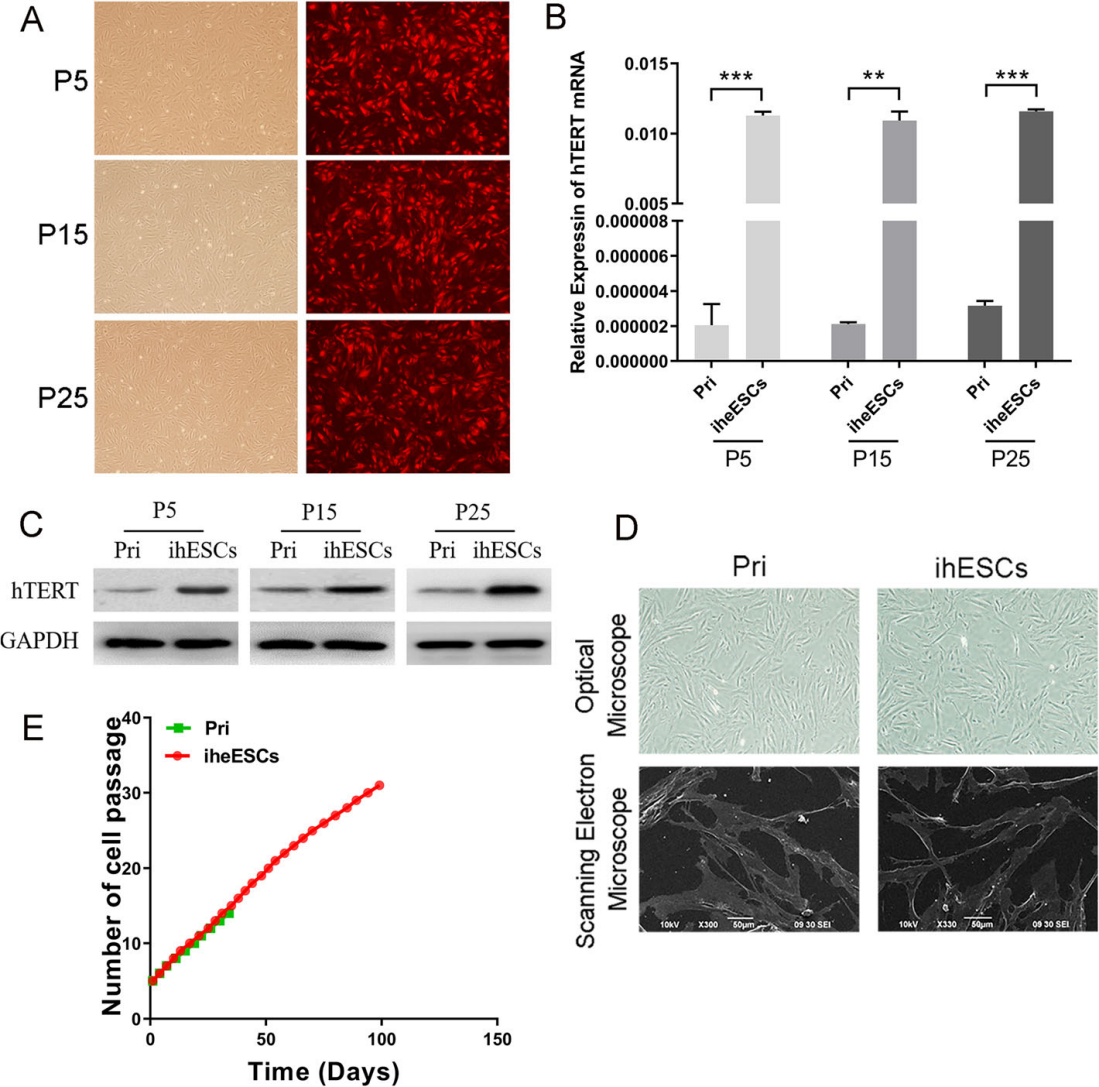
Establishment of immortalized human endometriotic stomal cells (ihESCs) by lentivirus transfection of hTERT. **a** The observation of red fluorescent from infected cells to estimate infection efficiency. **b** The expression of hTERT mRNA in ihESCs of 5, 15 and 25 passages. **c** hTERT protein level of ihESCs from various passages detected by Western Blot. **d** The morphology of ihESCs observed by using Optical Microscope (upper) and Scanning Electron Microscope (lower). **e** The life span of primary cells and ihESCs. Data represent the mean ± SEM. **P < .001, ***P < .0001

Lentivirus was employed to increase the hTERT expression of primary endometriotic stromal cells. As shown in Fig. 1a, the red fluorescence protein (RFP), as a molecular tracer, was stably expressed in ihESCs of 5, 15 and 25 passages. Further investigations were employed to detected the expression of hTERT in ihESCs from different passages. In Fig. 1b and c, both mRNA and proteins of hTERT in ihESCs were significant increased compared with primary cells. Furthermore, there was not obvious cell morphological change between ihESCs and primary stromal cells observed by optical microscope (Fig. 1d **upper**) and scanning electron microscope (Fig. 1d **lower**). To record life span of ihESCs and primary cells, the results of continuous cell cultivation exhibited that the life span of ihESCs which reached 31 passages much longer than that of primary cells which is 14 passages (Fig. 1e).

### The cell physiology of ihESCs keeps unchanged compared with primary cells

**Fig. 2:**
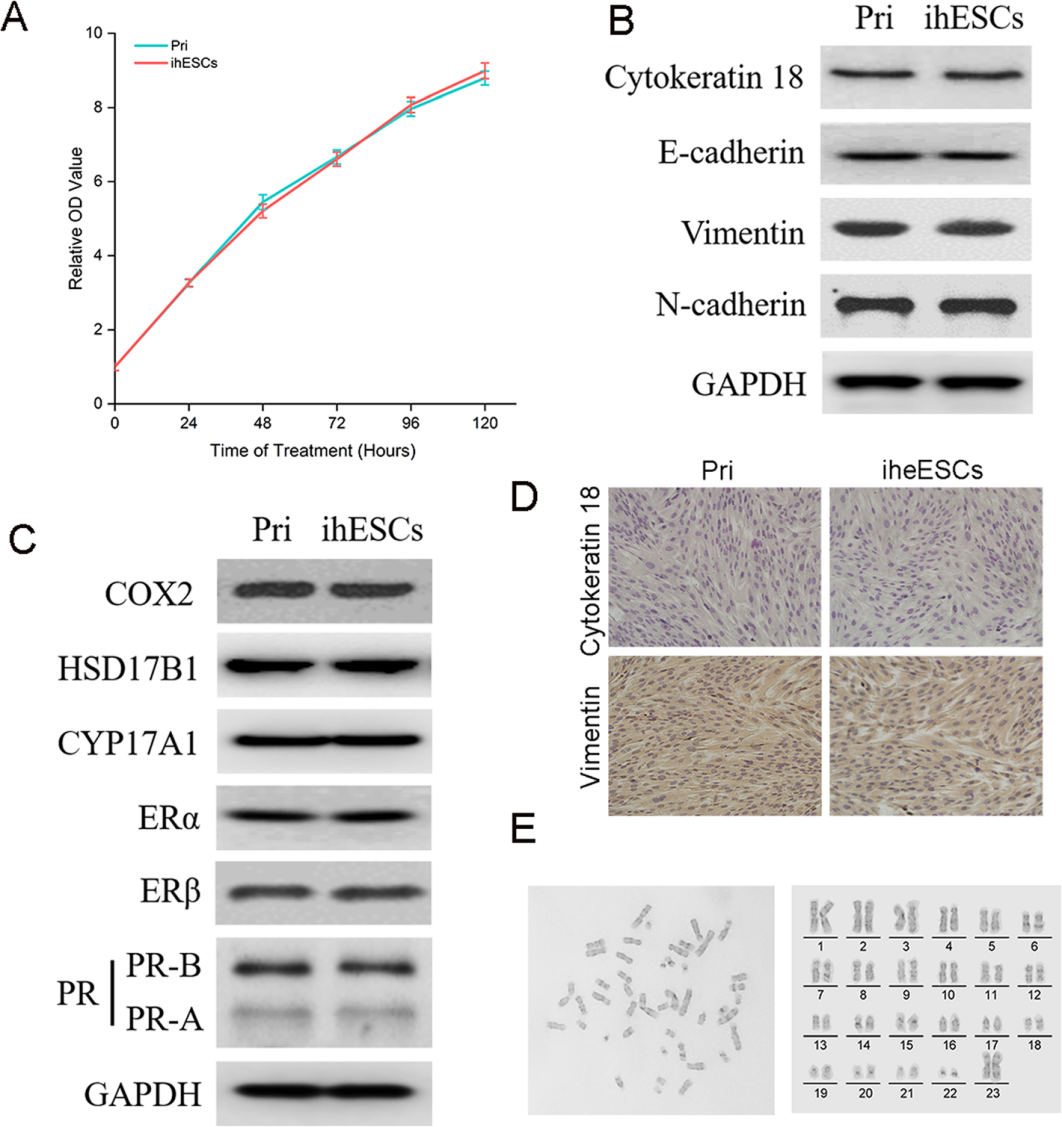
The phenotypes and marker proteins of ihESCs kept unchanged compared with primary stromal cells. **a** The growth curve of ihESCs and primary stromal cells detected by CCK-8 assays. **b** The protein expression of epithelial-mesenchymal transition from primary stomal cells and ihESCs. **c** The level of estrogen-metabolizing proteins and estrogen/progesterone receptors proteins in primary cell and ihESCs. **d** Identification of epithelial and mesenchymal cells assessed by immunocytochemistry (ICC). **e** Chromosome karyotype analysis of ihESCs and sorting result (right)

To further explore whether the cell physiology of ihESCs was changed, the proliferation and different marker proteins were detected. As in Fig. 2a, the proliferative activity of ihESCs extraordinary resembled primary cells. The expression of epithelial-mesenchymal transition (EMT) markers in ihESCs was consistent with primary cells (Fig. 2b). Furthermore, the estrogen-metabolizing proteins, including COX-2, HSD17B1 and CYP17A1, kept similar with primary cells. The expression of ERs and PRs were original level as well (Fig. 2c). Further, ihESCs maintain high expression of Vimentin and low expression of Cytokeratin 18, which suggested the mesenchymal cell characteristics of ihESCs were tenacious (Fig. 2d). To further examine the chromosomes of ihESCs, karyotype analysis was performed. As in Fig. 2e, ihESCs presented normal number of 23 pairs chromosomes.

### Normal estrogen response is kept in ihESCs

**Fig. 3:**
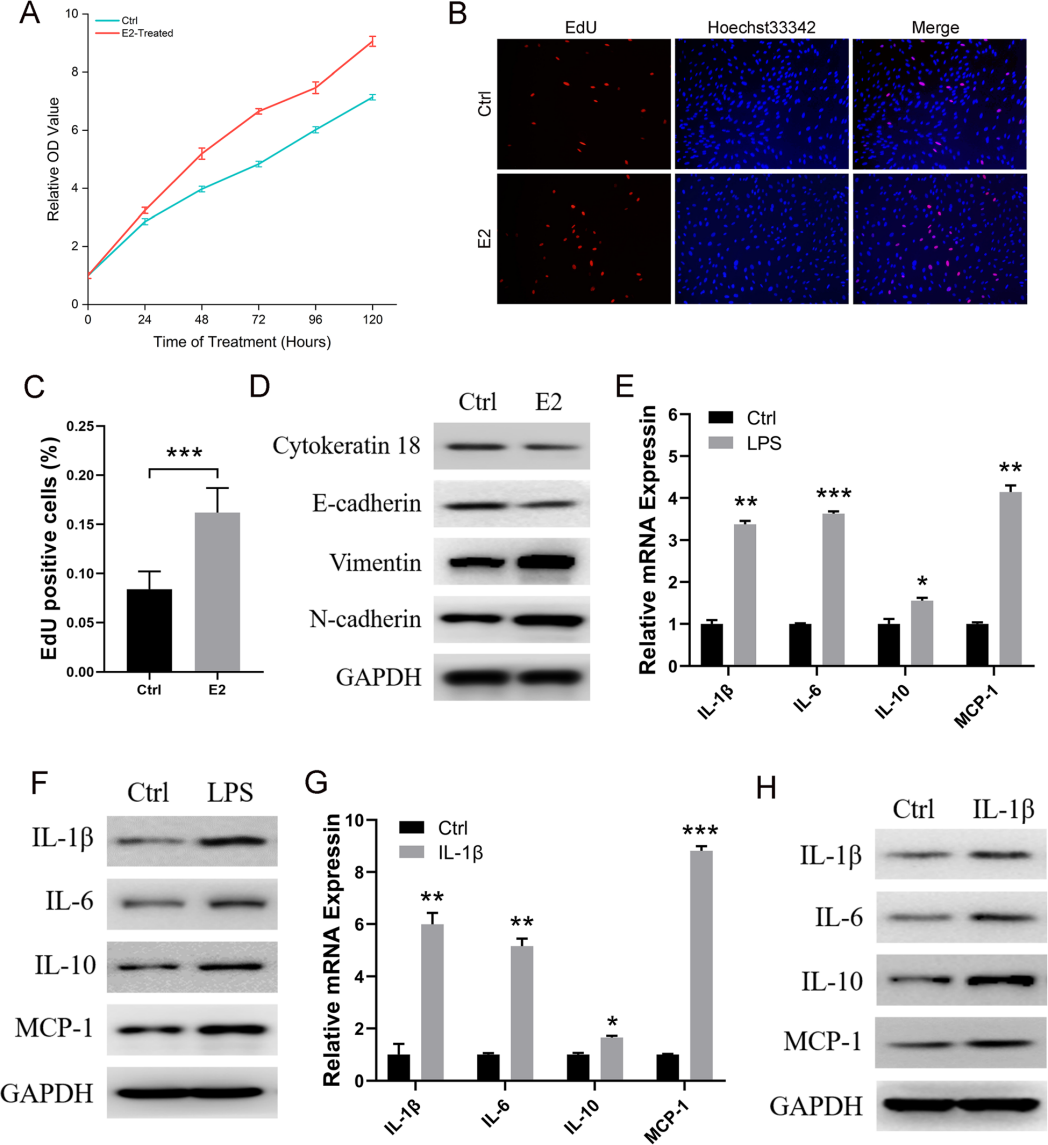
Normal estrogen response and inflammation are found in ihESCs. **a** The proliferation of ihESCs with stimulation of estrogen. **b** EdU-labeled assay employed to monitor cell division of ihESCs treated with estrogen. **c** Quantified analysis of EdU assays. **d** The protein expression of EMT markers from ihESCs with treatment of estrogen. The mRNA (**e**) and protein (**f**) expression of inflammatory factor in ihESCs with stimulation of LPS. The mRNA (**g**) and proteins (**h**) of inflammatory factor from ihESCs treated with IL-1β. Data represent the mean ± SEM. *P < .05, **P < .001, ***P < .0001

To demonstrate the response of ihESCs to estrogen stimulation, 200 nM estrogen was used to treat ihESCs. As shown in Fig. 3a, the treatment of estrogen significant promoted the proliferation of ihESCs. And stimulation of estrogen to ihESCs increased the number of EdU-labeled cells, which suggested estrogen could enhance cell division of ihESCs (Fig. 3b and c). Furthermore, estrogen reduced the expression of Cytokeratin 18 and E-cadherin proteins, but enhanced the level of mesenchymal marker proteins (Fig. 3d). Thus, estrogen promoted the proliferation and EMT of ihESCs, which suggested ihESCs kept normal response to estrogen.

### Inflammatory response is detected in ihESCs

To verify inflammatory response of ihESCs, 100 ng/ml LPS and 10 ng/ml IL-1β were employed to induced inflammation respectively. As the results of Fig. 3e and f, LPS remarkably promoted the expression of IL-1β, IL-6, IL-10 and MCP-1. And the mRNA and proteins of these inflammatory factors were significant increased with treatment of IL-1β (Fig. 3g and h). Therefore, the normal inflammatory response was retained in ihESCs.

### ihESCs are not available with tumorigenic ability

**Fig. 4:**
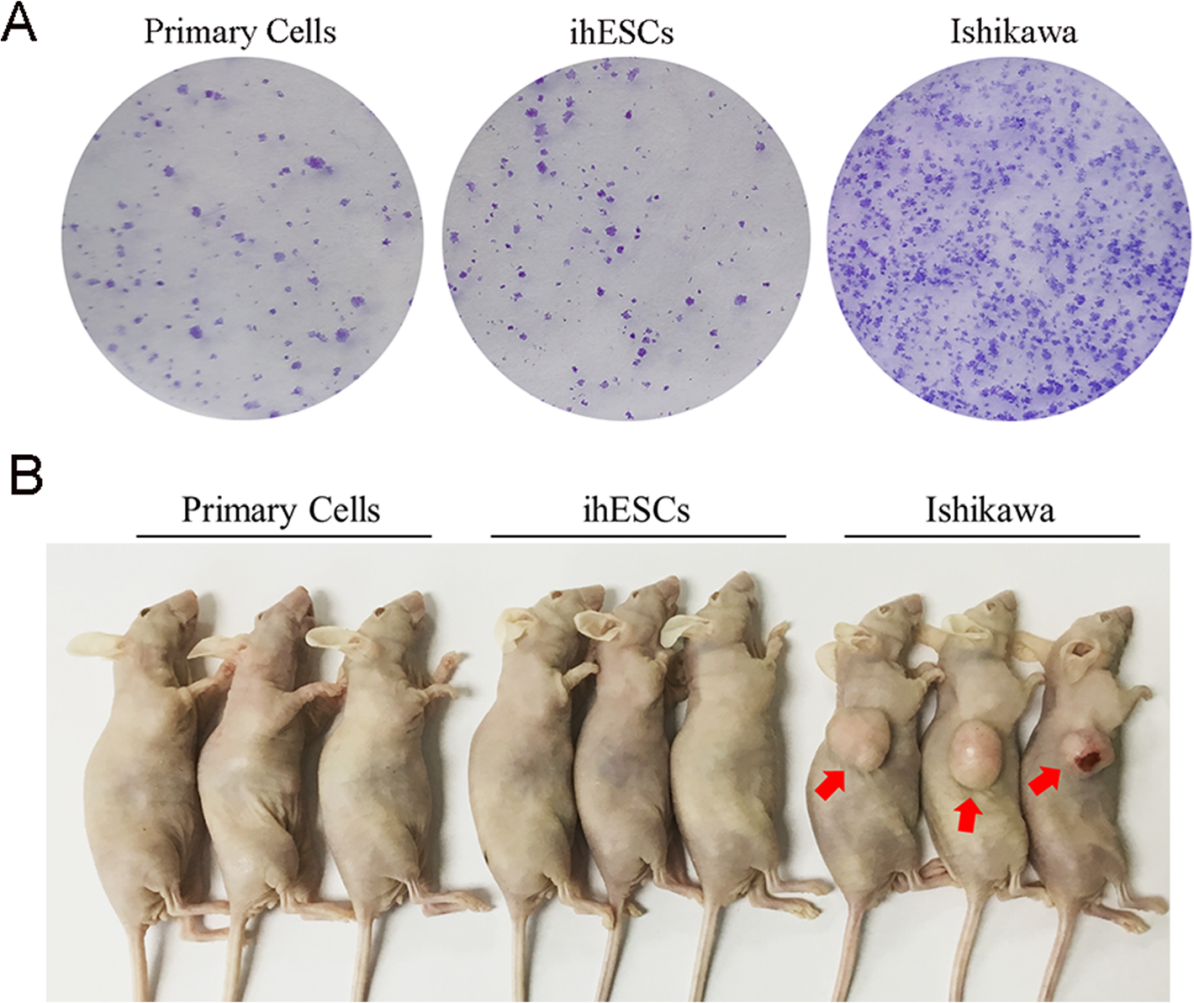
ihESCs are not malignant transformed to trigger tumors. **a** Colony formation assay used to assess the oncogenic potential of ihESCs. **b** Nude mouse tumorigenicity assay performed to evaluate tumorigenic capacity of ihESCs

To identify whether ihESCs were malignant transformation, colony formation assay and nude mouse tumorigenicity assay were performed. As shown in Fig. 4a, the clonogenesis ability of ihESCs was as weak as primary cells which much lower than endometrial cancer Ishikawa cells. Moreover, different from Ishikawa cells which induced formation of tumors, both ihESCs and primary stromal cells were incapable of triggering occurrence of tumors (Fig. 4b). Consequently, ihESCs kept characteristics of primary cells and were not malignant transformed.

## Discussions

This study established immortalized human endometriotic stromal cells (ihESCs) by overexpressing hTERT. The lifespan of immortalized cells was beyond 30 passages which much longer than primary cells. The morphology and proliferation of ihESCs remained original. The expression of EMT markers, estrogen-metabolizing proteins and estrogen/progestogen receptors in ihESCs were unchanged from the primary cells. The chromosome karyotype of ihESCs retained normal. The normal feedback of estrogen and inflammation response were observed in ihESCs. Furthermore, ihESCs were not malignant transformed to trigger the formation of tumors. Thus, we established an immortalized endometriotic stromal cells with stable characteristics and function, which might be a practical cellular tool.

According to abundant reports about high-throughput sequencing of endometriosis, thousands of genes aberrant expressed in endometriosis were found [20–22]. Compared with normal and eutopic endometria, endometriotic lesions were remarkably mutative including change of inflammation response [13, 23], hormone dysregulation [14], disordered proliferation and apoptosis [24, 25], promoted angiogenesis [26] and EMT [27]. Therefore, endometriotic cells are much more appreciate model cells in the mechanism research of endometriosis. To establish model cells with stabled characteristics and long lifespan is considerable significance for practical application. Thus, establishment of immortalized endometriotic cells could be an effective method to supply the cell tool for research. The immortalized cell ihESCs established in this study remained unchanged morphology, proliferation and EMT markers, which indicated that the process of immortalization did not affect the growth and type of cells. On the other hands, it was well recognized that estrogen was greatly increased in endometriotic lesions compared with normal and eutopic endometria [14]. And significantly increased estrogen synthases are closely associated with recurrence of endometriosis [28–30]. Our results manifested that these estrogen synthases including COX-2, HSD17B1 and CYP17A1 kept consistent with primary cells. Furthermore, the expression of estrogen/progestogen receptors were not changed, which implied that the functional approaches of estrogen and progestogen remain complete. Moreover, the immortalized cells maintained normal number of chromosomes suggesting the genes were not obviously changed with the influence of immortalization. Therefore, these results demonstrated that ihESCs kept morphology and genes characteristics as the primary endometrial stromal cells.

Endometriosis is recognized as estrogen-dependent and inflammatory disorder [29, 30]. Estrogen stimulated the proliferation of endometriotic cells predominantly via its nuclear receptors [31]. And our previous study found that estrogen markedly promoted EMT [32]. In this study, the proliferation and EMT of ihESCs were obviously promoted with stimulation of estrogen, which suggested the original ability responding to estrogen of primary cell remained in ihESCs. Additionally, the treatment of LPS and IL-1β could trigger obvious inflammation of ihESCs, which demonstrated that inflammatory response was present in ihESCs. Consequently, ihESCs suffice for the usage of research about the effect and mechanisms of estrogen and inflammation on endometriosis.

A previous study analyzed numerous genes expression associated with estrogen/progesterone biosynthesis and signaling, cell cycle regulation, and cytokine production from different immortalized human endometriotic epithelial cells and stromal cells [33]. Mariarosaria et all established immortalized endometriotic epithelial and stromal cells by retroviral transfection of hTERT, which kept the primary characteristics of phenotype and expression of estrogen and progesterone receptors [34]. However, the change of cell morphology, physiology and malignant transformation were lack of identification in these immortalized cells. To evaluate comprehensively our immortalized cell, the cell morphology, proliferation, EMT markers and different cell physiology were monitored unchanged. Moreover, the karyotype analysis and tumor formation experiment demonstrated that ihESCs were not malignant transformed and could be reliable model cells. In conclusion, this study established immortalized endometriotic stromal cells, and characterized that these cells kept much longer lifespan, primary physiological characteristics and were not malignant transformed. Thus, ihESCs are potentially useful as an experimental model to demonstrate endometriosis.

## Data Availability

All data in this study are included in this published article.

## Abbreviations

ihESCs: Immortalized human endometriotic stromal cells (ihESCs)
hTERT: Human telomerase reverse transcriptase
qRT-PCR: Quantitative real-time polymerase chain reaction
EMT: Epithelial-mesenchymal transition
LPS: Lipopolysaccharide
E2: Estrogen
